# Upregulation of pirin expression by chronic cigarette smoking is associated with bronchial epithelial cell apoptosis

**DOI:** 10.1186/1465-9921-8-10

**Published:** 2007-02-08

**Authors:** Brian D Gelbman, Adriana Heguy, Timothy P O'Connor, Joseph Zabner, Ronald G Crystal

**Affiliations:** 1 Division of Pulmonary and Critical Care Medicine, Weill Medical College of Cornell University, New York, New York, USA; 2 Department of Genetic Medicine, Weill Medical College of Cornell University, New York, New York, USA; 3 Pulmonary, Critical Care and Occupational Medicine, Department of Internal Medicine, University of Iowa, Iowa City, IA, USA

## Abstract

**Background:**

Cigarette smoke disrupts the protective barrier established by the airway epithelium through direct damage to the epithelial cells, leading to cell death. Since the morphology of the airway epithelium of smokers does not typically demonstrate necrosis, the most likely mechanism for epithelial cell death in response to cigarette smoke is apoptosis. We hypothesized that cigarette smoke directly up-regulates expression of apoptotic genes, which could play a role in airway epithelial apoptosis.

**Methods:**

Microarray analysis of airway epithelium obtained by bronchoscopy on matched cohorts of 13 phenotypically normal smokers and 9 non-smokers was used to identify specific genes modulated by smoking that were associated with apoptosis. Among the up-regulated apoptotic genes was pirin (3.1-fold, p < 0.002), an iron-binding nuclear protein and transcription cofactor. *In vitro *studies using human bronchial cells exposed to cigarette smoke extract (CSE) and an adenovirus vector encoding the pirin cDNA (AdPirin) were performed to test the direct effect of cigarette smoke on pirin expression and the effect of pirin expression on apoptosis.

**Results:**

Quantitative TaqMan RT-PCR confirmed a 2-fold increase in pirin expression in the airway epithelium of smokers compared to non-smokers (p < 0.02). CSE applied to primary human bronchial epithelial cell cultures demonstrated that pirin mRNA levels increase in a time-and concentration-dependent manner (p < 0.03, all conditions compared to controls).

Overexpression of pirin, using the vector AdPirin, in human bronchial epithelial cells was associated with an increase in the number of apoptotic cells assessed by both TUNEL assay (5-fold, p < 0.01) and ELISA for cytoplasmic nucleosomes (19.3-fold, p < 0.01) compared to control adenovirus vector.

**Conclusion:**

These observations suggest that up-regulation of pirin may represent one mechanism by which cigarette smoke induces apoptosis in the airway epithelium, an observation that has implications for the pathogenesis of cigarette smoke-induced diseases.

## Background

Although the airway epithelium has several defense mechanisms that respond to the stress imposed by cigarette smoke, in some individuals these defenses are exaggerated, resulting in chronic inflammation, and eventually, chronic bronchitis [1-5]. One of the earliest effects of cigarette smoke on the airway epithelium is to disrupt the protective barrier that is normally mediated by tight junctions between epithelial cells, resulting in increased permeability across the epithelium [6-8]. The disruption of the epithelial barrier is associated with an innate immune response, typified by the migration of inflammatory cells into the epithelial layer [3,4]. If this inflammatory response, becomes chronic, it eventuates into the airway pathology and dysfunction associated with chronic bronchitis [9].

The mechanism by which the bronchial epithelium becomes disrupted by cigarette smoke is not fully understood, but evidence suggests that damage to the epithelial cell *per se *is more important than dysfunction of the junctions between epithelial cells [10,11]. Since the morphology of the airway epithelium of cigarette smokers does not typically demonstrate epithelial necrosis, the most likely mechanism for epithelial cell death in response to cigarette smoke is apoptosis [12]. Consistent with this concept, *in vitro *studies have shown that exposure to cigarette smoke can initiate apoptosis in fibroblasts, macrophages and alveolar epithelial cell lines [13-15], and apoptosis of airway epithelial cells has been observed in experimental animals in response to cigarette smoke [16,17], but *in vitro *studies of bronchial epithelial cells exposed to cigarette smoke have yielded conflicting results [18-20].

In the context of these considerations, we hypothesized that cigarette smoke modulates the expression of genes in the airway epithelium that initiate the cells to undergo apoptosis. To assess this hypothesis, gene expression profiling was used to determine if cigarette smoking in humans is associated with the up-regulation of pro-apoptotic genes (or the down-regulation of anti-apoptotic genes) in the bronchial epithelium. To obtain the relevant biologic samples, we used fiberoptic bronchoscopy with airway brushing to sample the bronchial epithelium from smokers and non-smokers. To avoid the confounding effects of already established lung disease, airway epithelium was sampled from phenotypically normal ~20 pack-yr smokers. The genes that were up-regulated in the smokers were functionally annotated using public databases according to their potential role in apoptosis. Pirin, a transcription cofactor that has been shown in eukaryotic cells to be induced during apoptosis [21], was selected for further study because it had the highest fold change in expression level in association with smoking. *In vitro *experiments in which primary and transformed human airway epithelial cell lines were exposed to cigarette smoke extract confirmed that cigarette smoke *per se *up-regulated pirin mRNA levels. Using an adenovirus gene transfer vector that constitutively expressed pirin, studies in a bronchial epithelial cell line showed that pirin up-regulation was associated with apoptosis. Together, the data suggests that cigarette smoking up-regulates pirin expression in the bronchial epithelium, with an associated increase in apoptosis, thus identifying at least one signaling mechanism associated with the disruption of the airway epithelial barrier in cigarette smokers.

## Methods

### Study Individuals

Healthy non-smokers and healthy chronic smokers were recruited using local print advertisements. The study individuals are part of an ongoing project to assess gene expression in the human airway epithelium in regard to the chronic airway disorders associated with cigarette smoking [22-24]. The study was approved by the Weill Cornell Medical College Institutional Review Board and written informed consent was obtained from each individual before enrollment in the study. The smokers had an approximate smoking history of 20 pack-yr and were in otherwise good health, with no evidence of respiratory tract infection, chronic bronchitis or lung cancer. Each individual had to complete an initial screening evaluation, which included a history of smoking habits, respiratory tract symptoms, and prior illnesses, a complete physical exam, chest radiograph, and pulmonary function tests. Routine screening blood and urine studies were performed, including urinary levels of nicotine and its derivative cotinine, and serum levels of carboxyhemoglobin to verify reported levels of smoking.

### Collection of Airway Epithelial Cells

All individuals who met the inclusion and exclusion criteria underwent fiberoptic bronchoscopy with brushing of the 3^rd ^to 4^th ^order bronchi as previously described [22]. Smokers were instructed not to smoke the evening before undergoing bronchoscopy. A 1 mm disposable brush (Wiltek Medical, Winston-Salem, NC) advanced through the working channel of the bronchoscope was used to collect the airway epithelial cells by gently gliding the brush back and forth on the airway epithelium 5 to10 times in 10 different locations in the third branching of the bronchi in the right and left lower lobe of each individual. The cells were detached from the brush by flicking it into 5 ml of ice-cold LHC8 medium (GIBCO, Grand Island, NY). An aliquot of 0.5 ml was kept for differential cell count and for cytology; the remainder (4.5 ml) was processed immediately for RNA extraction. Total cell number was determined by counting on a hemocytometer. Differential cell count (epithelial *vs *inflammatory cells) was assessed on cells prepared by cytocentrifugation (Cytospin 11, Shandon Instruments, Pittsburgh, PA) stained with DiffQuik (Baxter Healthcare, Miami FL).

### Preparation of cDNA and Hybridization to Microarray

All analyses were performed using the Affymetrix HuGeneFL chip and associated protocol from Affymetrix (Santa Clara, CA). Total RNA was extracted from brushed cells using TRIzol (Life Technologies, Rockville, MD) followed by RNeasy (Qiagen, Valencia CA) to remove residual DNA, which yielded approximately 2 μg RNA from 10^6 ^cells. First strand DNA was synthesized using the T7-(dT)_24 _primer and converted to double stranded cDNA using Superscript Choice system (Life Technologies). cDNA was purified by phenol chloroform extraction and precipitation, and the size distribution was examined after agarose gel electrophoresis. The cDNA was then used to synthesize biotinylated RNA transcript using the Bioarray HighYield reagents (Enzo, New York, NY). This was purified by RNeasy (Qiagen) and fragmented immediately before use. The labeled cRNA was hybridized to the HuGeneFL GeneChip for 16 hr, and then processed by the fluidics station under the control of Microarray suite software (Affymetrix). The chip was then manually transferred to the scanner for data acquisition.

### TaqMan RT-PCR

RNA levels for pirin were measured relative to 18s rRNA by real time quantitative PCR (TaqMan) with fluorescent TaqMan chemistry using the ΔΔCt method (PE Biosystems, Instruction Manual). TaqMan reactions for pirin were optimized and validated to show equal amplication efficacy compared to 18s rRNA using adult human lung RNA (Strategene, La Jolla, CA). Two sets of primers and probes were used, one to measure endogenous RNA (including 3' untranslated end), and one to measure both endogenous and adenovirus-produced pirin mRNA (which spans two exons and would not amplify genomic DNA). The endogenous specific pirin primers were: forward AATGGGTTTGAAAGGGCCA and reverse TCAAGACCTGCTCTTCCGCT, with probe AACCTGGAAATCAAAGATTGGGAACTAGTGGA. The endogenous and adenovirus-produced pirin primers were: forward CACGCTGAGATGCCTTGCT and reverse ACCATCTTCTCTGAGCTCCTCAA with probe CAGCCCATGGCCTACAACTGTGGGTTATA.

### Exposure of Primary Human Bronchial Epithelial Cells to Cigarette Smoke Extract

Three separate primary human bronchial epithelial (HBE) cell cultures were isolated from trachea and bronchi of donor lungs and seeded onto collagen-coated semi-permeable membranes (Millipore, Bedford, MA) and grown at the air-liquid interface [25]. The viability of the cells was confirmed before each experiment by measurement of transepithelial resistance [25].

Cigarette smoke extract (CSE) was prepared using a modification of the method used by Wyatt et al [26]. Four research grade cigarettes (2R4F, University of Kentucky) were bubbled into 50 ml of 1:1 DMEM:Ham F12 medium using a vacuum pump apparatus. The CSE was filtered through a 0.22 μm filter to remove particles and bacteria before use. Solutions of 10% and 100% CSE were prepared from this stock. The solution of CSE (15 μl) was applied to the apical surface of the HBE cells and RNA was isolated from the cultures at 2, 24, and 48 hr after CSE exposure using TRIzol (Life Technologies) followed by RNeasy (Qiagen). Samples were obtained in triplicate for each time point. Pirin RNA levels were measured by TaqMan RT-PCR using the primers and probe described above. Pirin expression levels relative to 18s rRNA were assessed. Each data point was generated from triplicate wells for each of the three separate cell lines.

### Assessment of Pirin-induced Apoptosis

To assess the relationship between up-regulation of pirin and the induction of apoptosis, an adenovirus (Ad) gene transfer vector coding for pirin was used to transfer the human pirin cDNA to human bronchial epithelial cells, and pirin expression and apoptosis were assessed over time. The recombinant Ad vectors AdPirin and AdNull used in this study are E1a^-^, partial E1b^-^, and partial E3^-^, based on the Ad5 genome, with the expression cassette in the E1 position [27-29]. The AdPirin expression cassette includes the cytomegalovirus early/intermediate enhancer/promoter (CMV), an artificial splice signal, the human pirin cDNA (obtained from A549 cells), and an SV40 stop/poly (A) signal. The AdNull vector is identical to the AdPirin vector, except that it lacks a cDNA in expression cassette [28]. The vectors were propagated, purified, and stored at -70°C [27].

AdPirin-induced apoptosis was assessed in the human airway epithelial BEAS-2B cell line [30]. BEAS-2B cells (ATCC, Rockville, Maryland) were grown on lysine coated coverslips in LHC-9 medium (Biosource International), Camarillo, CA) until they were 50 to 60% confluent. The cells were then infected with AdNull and AdPirin at varying concentrations [10^3 ^and 10^4 ^particle units (pu)].

Two assays were used to assess apoptosis: TdT-mediated dUTP nick end labeling (TUNEL) assay and cytoplasmic nucleosome ELISA. For the TUNEL assay, cells were fixed to the coverslips using 4% paraformaldehyde and then permeabilized with 0.2% Triton X-100 in PBS. Cells were equilibrated with equilibration buffer, nucleotide mix, and rTdT enzyme (Promega, Madison WI) for 60 min and then washed. DAPI nuclear counterstain was applied before cells were mounted onto slides and evaluated under fluorescent microscope. The percentage of apoptotic cells per 10× field were manually counted in 10 fields per slide. For the cytoplasmic nucleosome ELISA assay (Cell Death Detection ELISA, Roche, Indianapolis, IN), BEAS-2B cells were lysed with lysis buffer, centrifuged for 10 min at 200 × g to pellet nuclei. The supernatant (20 μl) was added to the immunoreagent containing anti-histone biotin and anti-DNA horseradish peroxidase (HRP). Sample wells were placed on shaker at 300 rpm for 2 hr, 23°C. 2, 2azino-di[3-ethylbenzthiazolin-sulfonate] (ABTS) solution was added and photometric analysis was measured at 405 nm, subtracted from background 490 nm. For each sample, the fluorescent value was normalized to the internal negative control of the experiment to generate an apoptotic index, which reflects the fold change in the number of apoptotic cells for experimental condition compared to control.

### Northern Analysis

A ^32^P-labeled pirin specific DNA probe was synthesized using strip EZ labeling kit (Roche, Indianapolis, IN) from a pirin cDNA template amplified from human genomic DNA. RNA electrophoresis was performed in 1% agarose gel followed by transfer to nitrocellulose membrane and UV crosslinking. The membrane was then hybridized with DNA probe and exposed to X ray film for 1 hr.

### Statistical Analyses

The microarray data was analyzed using the GeneSpring software (Silicon Genetics, Redwood City, CA). Normalization was carried out sequentially: per microarray sample (dividing the raw data by the 50th percentile for all measurements) and then per gene (dividing the raw data by the median of the expression levels for the given gene in all samples). Data from probe sets representing genes that failed the Affymetrix detection criteria (labeled "Absent" or "Marginal") in all 44 microarrays were eliminated from further analysis. The p value for each gene was calculated comparing the non-smokers with smokers using the Welch t-test with a Benjamini-Hochberg correction for false discovery rate. For the *in vitro *studies, comparisons between RNA expression levels for pirin and percentage of apoptotic cells were made using the Student's two-tailed t-test.

## Results

### Microarray Analysis

The microarray analysis was carried out in a data set previously reported using a total of 44 Affymetrix HuGene FL microarrays to assess left and right samples from 22 individuals, including 9 non-smokers and 13 smokers [22,24]. These 44 microarrays passed quality control as assessed by the GeneSpring software (Silicon Genetics, Redwood City, CA). The smokers and non-smokers were comparable with respect to yield and percentage of non-epithelial cells. To eliminate genes not expressed in airway epithelium, or expressed at low levels, those genes that were called absent by the Microarray Suite software (Affymetrix) in all of the 44 microarrays were discarded before further analysis. The number of genes remaining (i.e., expressed on at least 1 of the 44 microarrays) was 4,512. Using this subset of genes, non-parametric statistical methods (GeneSpring software) were used to identify genes which were expressed at a higher or lower level in a significant number of smokers *vs *non-smokers. Of the 4,512 genes that were expressed, there were a total of 85 probesets that were significantly (p < 0.05) up-regulated and 13 probesets down-regulated in smokers compared to non-smokers. The 98 probesets were functionally annotated by manual review of public databases (e.g. Medline, Locuslink) into categories that described their cellular processes. Of these, 7 of the up-regulated genes were identified to be associated with apoptosis, including pirin, retinoic acid receptor responder 2, prostrate differentiation factor, insulin-like growth factor binding protein 5, bone morphogenic protein 7, carcinoembryonic antigen-related cell adhesion molecule 6, and S100 calcium-binding protein A10 (Table 1). Of these, only two (retinoic acid receptor responder 2 and insulin-like growth factor binding protein 5) had any known association with cigarette smoke exposure. All of the genes identified were involved in signal transduction and transcription factors. Pirin, which has been shown to be induced during stress to cause cell death [21,31], was selected for further study because it had the highest fold change (3.12 smokers/non-smokers) amongst genes in this category.

**Table 1:** Apoptosis-relevant Genes Up-regulated in the Airway Epithelium of Smokers^1^

**Gene ID**	**Description**	**Smokers/non-smokers (fold up)**	**p value**	**Reference relevant to apoptosis**
Y07867	pirin	3.12	0.002	[21]
U77594	retinoic acid receptor responder 2	2.59	0.007	[39]
AB000584	prostate differentiation factor/growth differentiation factor 15	2.44	0.033	[40]
L27559	insulin-like growth factor binding protein 5	1.94	0.038	[41]
X51801	bone morphogenetic protein 7 (osteogenic protein 1)	1.83	0.046	[42]
M18728	carcinoembryonic antigen-related cell adhesion molecule 6	1.76	0.008	[43]
M38591	S100 calcium-binding protein A10	1.75	0.043	[44]

^1^All up-regulated genes were evaluated for an association with apoptosis by reviewing published information about each gene available in public databases. Genes that had experimental evidence linking their expression to apoptosis were selected for further study.

### Up-regulation of Pirin in Cigarette Smoker's Bronchial Epithelium *In Vivo*

**Figure 1:**
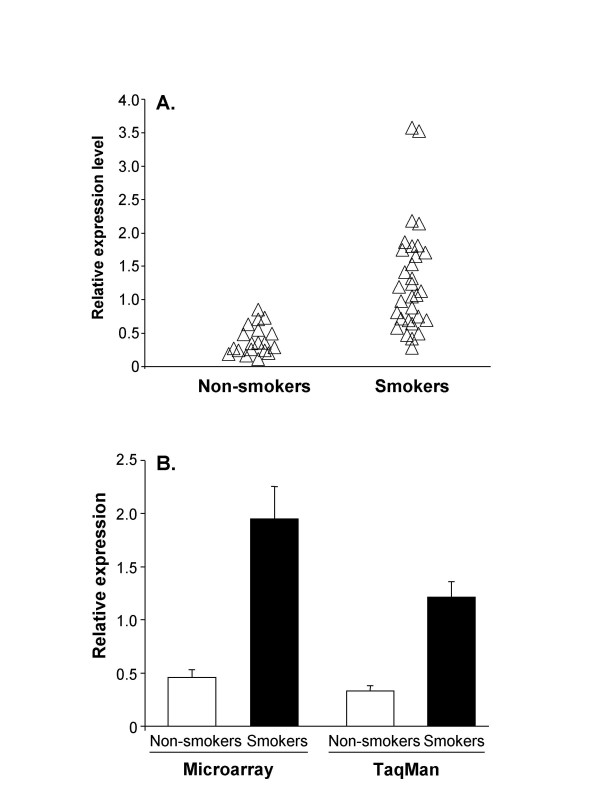
Mean RNA expression levels for pirin in smokers and non-smokers **A**. Microarray relative expression values (non-smokers n = 9, smokers n = 13, right and left lung samples for each individual). The relative expression level for pirin represents the normalized expression level in each array divided by the median expression level for pirin in all 44 microarrays. **B**. Comparison of microarray and TaqMan relative expression values (nonsmokers n = 3, smokers n = 9). Shown are the mean expression level of pirin ± standard error in airway epithelium of nonsmokers and smokers quantified by either microarray (p < 0.01) and TaqMan (p < 0.01).

The microarray data demonstrated (n = 18 samples from n = 9 non-smokers, n = 26 samples from n = 13 smokers) a significant up-regulation of pirin in the airway epithelium in the smokers (p = 0.002; Table 1, Figure 1A). Pirin expression levels are reproducible in samples from the right and left lungs of individuals, with no significant difference in pirin expression levels in the right lung versus the left lung (p > 0.15). To confirm the microarray data showing overexpression of pirin in the airway epithelium of smokers, pirin expression levels were assessed by an independent method using a subset (n = 6 samples from n = 3 non-smokers, n = 18 samples from n = 9 smokers) of the RNA samples studied by microarray analysis, for which there was adequate amount of RNA available. TaqMan RT-PCR confirmed that expression levels were significantly different between the two groups, reinforcing the validity of the observation with the microarray analysis that pirin mRNA levels are markedly elevated in the airway epithelium of smokers compared to non-smokers (p < 0.01; Figure 1B).

### Pirin Gene Expression Following Cigarette Smoke Exposure *In Vitro*

**Figure 2:**
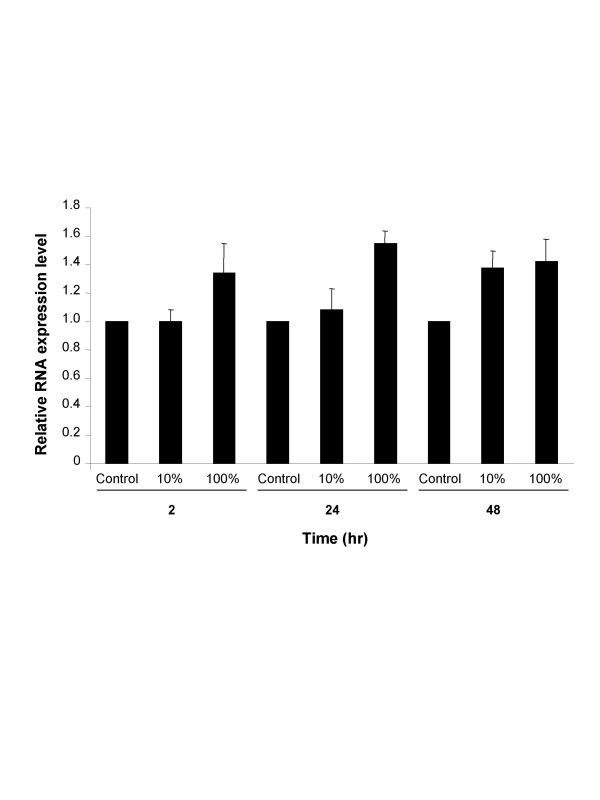
Mean RNA expression levels for pirin assessed by quantitative PCR in human bronchial epithelial (HBE) cells exposed to varying concentrations of cigarette smoke extract (CSE) over time. RNA was extracted from HBE cells after exposure to 10% and 100% CSE and pirin RNA levels were quantified relative to the control gene GAPDH by real-time quantitative PCR by TaqMan. Each data point was generated from triplicate wells; and a total of three separate data points were obtained for each condition at each time point. The ordinate represents the fold change in pirin expression in samples exposed to 10% and 100% CSE over time relative to the background pirin expression level in cells cultured in the absence of CSE.

To further establish that cigarette smoke up-regulates pirin expression in human bronchial epithelium, primary human bronchial epithelial cells were exposed to cigarette smoke extract *in vitro*. Human bronchial epithelial cells were used, as they most closely mimic airway epithelial cells in their natural environment *in vivo *[25]. TaqMan PCR with pirin RNA specific primers was used to quantify the amount of mRNA produced in the cells. Forty-eight hours after exposure to either 10% or 100% CSE, there was a respective 1.4-fold increase in pirin RNA levels in the cells exposed to cigarette smoke extract compared to the control group cultured in media (p < 0.03, 10% and 100% compared to controls; Figure 2).

### Induction of Bronchial Epithelial Cell Apoptosis in Association with Up-regulation of Pirin Expression

**Figure 3:**
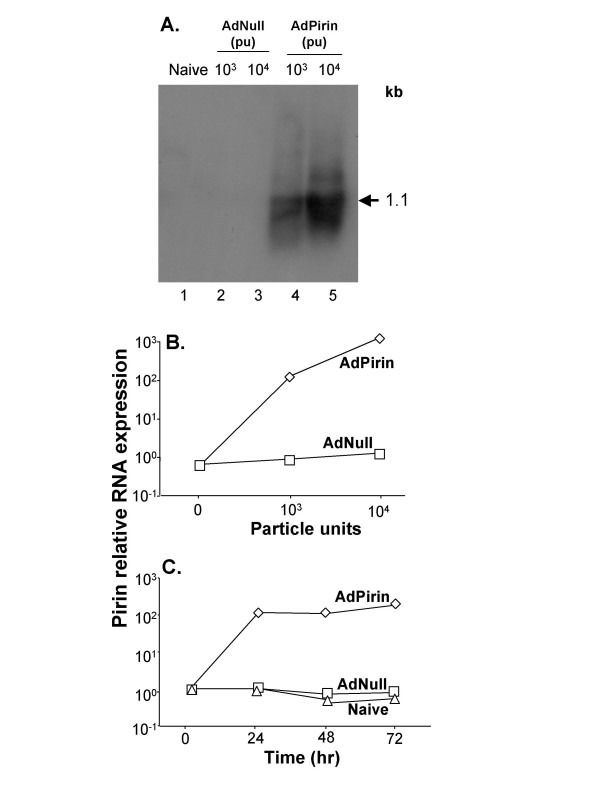
Mean RNA expression levels for pirin assessed by quantitative PCR in the human bronchial epithelial (BEAS-2B) cell line infected with AdPirin, AdNull, and negative control (naive) over varying concentrations (particle units, pu) and time. RNA was extracted from BEAS-2B cells that were infected with 10^3 ^and 10^4 ^particle units of AdPirin or AdNull. **A**. Northern analysis for Pirin RNA demonstrating the full length (1.1 kb) pirin mRNA directed by AdPirin 24 hr after infection. Lane 1 – control (naive); lane 2 – AdNull, 10^3 ^particle units; lane3 – AdNull, 10^4 ^particle units; lane 4 – AdPirin, 10^3 ^particle units; and lane 5 – AdPirin, 10^4 ^particle units. **B**. Concentration-dependent increase in pirin mRNA following infection with AdPirin. Pirin mRNA was quantified by TaqMan analysis. **C**. Time-dependent pirin mRNA expression over time evaluated by TaqMan.

**Figure 4:**
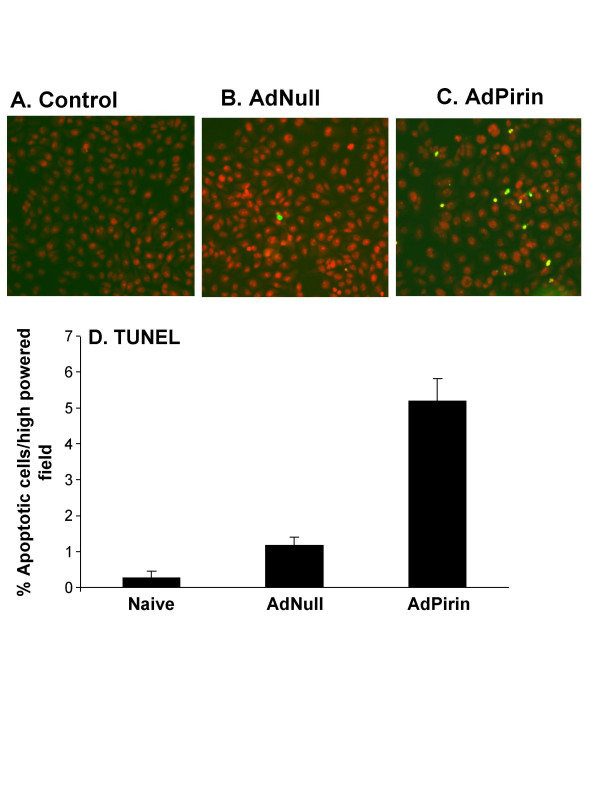
Apoptosis in BEAS-2B bronchial epithelial cells following up-regulation of pirin levels by infection with AdPirin. BEAS-2B cells were infected with AdPirin, AdNull, or control (naive) and TdT-mediated dUTP nick end labeling (TUNEL) was used to assess apoptosis after 24 hr. **A**. control (naive); **B**. AdNull (10^4 ^particle units); and **C**. AdPirin (10^4 ^particle units). **D**. Quantitative assessment of apoptosis in BEAS-2B bronchial epithelial cells exposed to AdPirin, AdNull or control by TUNEL assay. The percentage of apoptotic cells per 10× field were manually counted in 10 fields per slide, with three replicates.

To test the hypothesis that up-regulation of pirin expression is linked to increased apoptosis in epithelial cells, an adenovirus vector expressing human pirin cDNA was used to modify the human bronchial epithelial BEAS-2B cell line to express high levels of pirin RNA. Northern analysis demonstrated AdPirin-specific up-regulation of the 1.1 kb pirin mRNA (Figure 3A). TaqMan assessment of the pirin mRNA levels showed 79- and 538-fold change in expression at 10^3 ^and 10^4 ^particle units of AdPirin per cell, respectively (p < 0.01; Figure 3B). The expression of pirin was time independent, with expression up-regulated >100-fold for AdPirin at 24 to 72 hr (Figure 3C). Using TdT-mediated dUTP nick end labeling (TUNEL) to assess apoptosis, there was an approximately 5-fold increase in the number of TUNEL positive BEAS-2B cells exposed to 10^4 ^AdPirin compared to cells exposed to AdNull for 24 hr (p < 0.01; Figure 4A–D).

**Figure 5:**
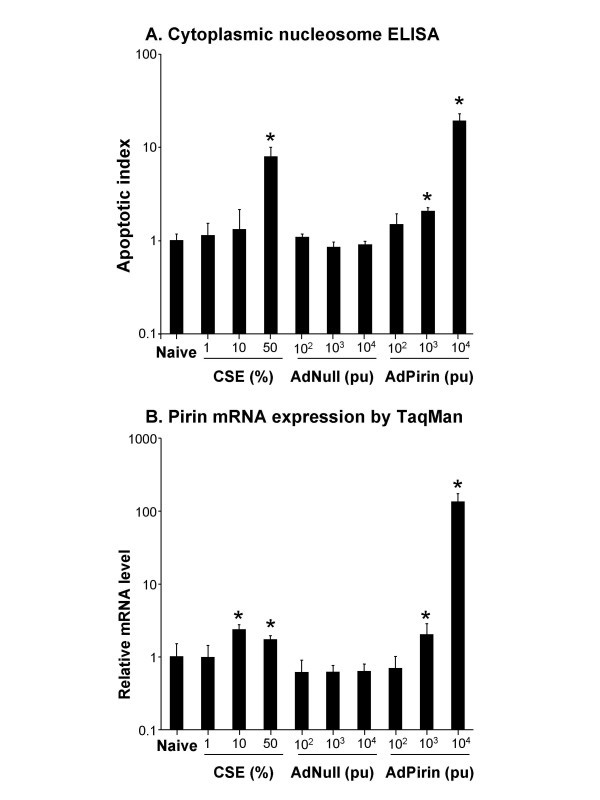
Evaluation of BEAS-2B bronchial epithelial cells exposed to varying concentrations of cigarette smoke extract, AdPirin and AdNull. **A**. Cytoplasmic nucleosome ELISA for apoptosis index. After cells were lysed and centrifuged to pellet nuclei, the supernatant was assessed by ELISA for the presence of cytoplasmic nucleosomes. The apoptotic index is the relative fluorescence value for given conditions, normalized to the naive control cells. Significant increases seen in apoptosis for cells exposed to 50% CSE, AdPirin (10^4 ^particle units) and AdPirin (10^3 ^particle units).**B**. Mean RNA expression levels for pirin assessed by quantitative TaqMan PCR in BEAS-2B cells exposed to varying concentrations of cigarette smoke extract (CSE), AdPirin and AdNull over time. The ordinate represents the fold change in pirin expression ± standard error in samples exposed to various conditions relative to the background pirin expression level in naive cells. Asterisk indicates a significant difference (p < 0.05) for conditions compared to naive control cells.

Confirmation of the TUNEL assay results were made using an ELISA against cytoplasmic nucleosomes. BEAS-2B cells were exposed to varying concentrations of AdPirin, AdNull and cigarette smoke extract (CSE) and evaluated for apoptosis and pirin RNA level. This assay, which uses an increase in fluorescent signal compared to the naive negative control to generate an apoptotic index, demonstrated a 19.3-fold increase for cells exposed to 10^4 ^AdPirin (p < 0.01), 2.1-fold increase for cells exposed to 10^3 ^AdPirin (p < 0.01) and 7.9 fold increase for cells exposed to 50% CSE (p < 0.01, Figure 5A). There was no significant increase in apoptotic cells compared to naive negative control for all other conditions. In this experiment, pirin RNA levels were increased 2.3 (p < 0.01) and 1.7-fold (p < 0.03) for BEAS-2B cells exposed to 10% and 50% CSE, respectively. BEAS-2B cells exposed to10^3 ^and 10^4 ^AdPirin demonstrated a 2.0 (p < 0.04) and 133.7-fold (p < 0.01) increase in pirin RNA expression level, respectively (Figure 5B).

## Discussion

Cigarette smoking is the major environmental exposure that leads to the pathogenesis of COPD [1-5]. The oxidative stress that cigarette smoke places on the airway epithelium results in a series of predictable morphologic changes over time. Despite our understanding of the pathologic changes that occur in the airways, the molecular mechanisms that direct the response of the airway epithelium to cigarette smoke are only partially understood [1-5]. The present study on pirin emerged from our laboratory's ongoing effort to identify unique cellular pathways that are linked to the development of lung diseases, such as COPD and lung cancer, by using gene expression analysis of the airway epithelium of smokers.

Apoptosis of airway epithelial cells is relevant to the pathogenesis of chronic bronchitis because disruption of epithelial integrity in the central airways appears to be an early event in response to cigarette smoke [6-8,32]. Based on the hypothesis that there may be apoptosis-related proteins expressed in the epithelial cells of cigarette smokers that have not been identified, we employed the unbiased strategy of assessing gene expression in airway epithelial cells of smokers *vs *non-smokers for the up-regulation of candidate proteins using microarray technology. Affymetrix gene chip assessment of airway epithelial cells from phenotypically normal ~20 pack-yr smokers compared to normal non-smokers demonstrated the up-regulation of several proteins that have been linked to apoptosis in the airway epithelium of smokers compared to non-smokers. Among the up-regulated proteins that were found to be associated with apoptosis was pirin, a nuclear transcription cofactor that is part of the cupin superfamily [21,33,34].

TaqMan real-time RT-PCR independently confirmed the microarray observed changes in pirin mRNA expression. To validate this *in vivo *observation, an *in vitro *model was designed to test the effect of cigarette smoke on pirin expression in epithelial cell culture. Using primary cultures of human bronchial epithelial cells, pirin expression was shown to respond to increasing concentrations of cigarette smoke extract relatively acutely, within 24 to 48 hr. A similar increase in pirin RNA expression level was observed in the BEAS-2B cell line when exposed to cigarette smoke extract. The potential for pirin to induce apoptosis in bronchial epithelial cells was assessed by using AdPirin, an adenovirus gene transfer vector expressing the human pirin cDNA, to over-express pirin in cultures of transformed airway epithelial cells. The data demonstrate that pirin over-expression results in significant epithelial cell apoptosis, a possible mechanism that leads to a breakdown of the epithelium in response to cigarette smoke. Although the magnitude of pirin over-expression is greater in the *in vitro *AdPirin experiments than that observed *in vivo*, it is important to recognize that the in vivo upregulation of pirin expression occurs after chronic smoking (~20 pack-yr), and this chronic, low level up-regulation over an extended period of time is impossible to emulate *in vitro*. It is therefore not surprising, that in order to demonstrate an *in vitro *effect on apoptosis over a much shorter time course (2–3 days), a higher expression of the gene was required. Interestingly, in the experiments where bronchial epithelial cells (BEAS-2B) were exposed to cigarette smoke extract, there was a similar 2 fold increase in pirin expression to 10% CSE and a significant increase in apoptosis, thus paralleling the *in vivo *observation.

### Apoptosis and the Pathogenesis of Chronic Bronchitis

One of the main functions of the airway epithelium is to provide a physical barrier to protect the underlying tissue from the harmful effects of cigarette smoke [1,2,4,5]. The inflammatory response seen in COPD is initiated when the airway epithelium is disrupted by chronic exposure to cigarette smoke [4]. This breach in epithelial integrity triggers the innate immune response which mobilizes neutrophils, eosinophils, macrophages, natural killer cells and mast cells into the subepithelial space [2,9]. When the stress of cigarette smoke is exerted over years, the persistent inflammation in the central airways leads to the pathologic finding of chronic bronchitis. As the inflammation extends to the small airways (between 4^th ^to 14^th ^generation bronchi), inflammatory exudates can accumulate in the lumen of the bronchi resulting in airflow obstruction [9].

Epithelial permeability, as measured by ^99m^TC-DTPA lung clearance in human subjects, has been shown to be increased in chronic smokers compared to non-smokers [7,32]. Studies using animal models have shown that epithelial cell permeability can increase in response to cigarette smoke with 30 min and 6 hr of exposure [8,11]. This is followed by an increased number of neutrophils and mast cells in the airway wall 6 hr after cigarette smoke exposure. The mechanism for increased permeability is unclear, but prior studies have shown that damage to the epithelial cell itself, and to a lesser extent, disruption in the tight junctions between epithelial cells are partly responsible [10,11]. Consistent with the bronchial epithelial cell studies, *in vitro *cellular studies have shown that alveolar epithelial cell viability decreases after exposure to cigarette smoke in a time and concentration dependent manner [35].

The mechanism of cell death for the epithelium in response to cigarette smoke may involve some necrosis, but apoptosis is likely the dominant mechanism [18]. Mouse models of smoking induced airway disease have demonstrated that apoptosis can occur in the airways in response to cigarette smoke [16,17]. Due to the efficient nature of the apoptotic pathway, allowing for rapid clearance of dying cells, it can be technically difficult to assess for apoptosis from *in vivo *studies of the airway in humans.

Several *in vitro *studies have shown that apoptosis can occur in response to cigarette smoke extract (CSE) in a variety of cell types, including airway epithelial cells, alveolar macrophages, endothelial cells, alveolar epithelial cells, and fibroblasts [13-15]. In contrast, Wickenden et al [19] found that CSE induced necrosis of a variety of cell types, while inhibiting the activation of the caspase system and preventing apoptosis. This discrepancy may be explained by the observation that at low concentrations, CSE induces apoptosis, while at higher concentrations, necrosis is induced [18]. Liu et al [20] reported that CSE caused DNA damage and initiated DNA repair processes, but not apoptosis, in bronchial epithelial (BEAS-2B) cells. The reason their conclusions differ from ours is likely due to differences in the concentration of cigarette smoke extract used. The maximum concentration that Liu [20] et al used was 10% CSE generated from 1 cigarette bubbled into sterile water. Similarly, in our experiment we did not observe apoptosis in BEAS-2B cells exposed to 10% CSE, but did observe apoptosis in cells exposed to 50% CSE, generated from 4 cigarettes bubbled into LHC-9 media. Taking all of the *in vitro *studies into consideration, one potential explanation is that at low concentrations, CSE causes DNA damage that is reparable until a threshhold concentration is reached, after which the cell initiates apoptosis. Very high levels of CSE likely cause overt necrosis.

### Induction of Apoptosis by Pirin

Pirin was first identified by Wendler et al [33], where it was found to interact with nuclear factor 1/CCAAT box transcription factor (NF1/CTF1). Pirin was found to be exclusively nuclear, expressed in most tissues and highly conserved among mammals. The N-terminus shares significant homologies with proteins from plants, fungi and prokaryotes.

Pirin was later found to bind to the oncoprotein B-cell lymphoma 3-encoded (Bcl-3), a member of the IκB family[36]. Bcl-3 interacts with the anti-apoptotic protein NF-κB, by binding to the p50 and p52 [36]. Recently, the crystal structure of pirin has been elucidated and was found to have a single Fe^2+ ^ion in the highly conserved N-terminal region [34]. This metal ion may be necessary for its interaction with Bcl-3 and NF-κB. Bcl-3 can either inhibit or enhance NF-κB DNA binding and transcription depending on its binding to other transcription co-factors.

Pirin stabilizes the formation of quaternary complexes between Bcl-3, NF-κB, and its DNA target. While it is not clear exactly how pirin influences the interaction between Bcl-3 and NF-κB, based on our results and others, it seems possible that pirin directs NF-κB DNA binding towards a pro-apoptotic response. Elucidating this mechanism will require further investigation to understanding the signaling that results from pirin expression. This may not be possible through gene expression studies alone, since several apoptotic pathways are induced by mechanisms other than up-regulation of mRNA levels of pro-apoptotic genes. This might explain why there was no observed upregulation of traditional apoptotic or inflammatory pathway genes, e.g., caspases and Bcl-2, in our microarray data obtained from human airway epithelium. The lack of up-regulation of pro-inflammatory genes in the airway epithelium of smokers is also supported by the transcriptome analysis of Spira et al [37]. One possible explanation for this observation is that many of these inflammatory mediators may be released primarily by non-epithelial cells.

Evidence for pirin involvement in apoptosis comes from experiments in tomatoes [21]. When tomato cells in suspension are treated with the topoisomerase inhibitor, campothecin, a potent initiator of cell death, *le-pirin*, an ortholog to human pirin, mRNA levels are dramatically increased. In the cyanobacterium *Synechocystis*, pirin orthologs are highly induced under conditions of severe salt stress [28].

We originally noted pirin to be up-regulated in microarrays of phenotypically normal individuals that smoke [24], a finding also made by Spira et al [37], in their study of genes up-regulated in the airway epithelium of smokers. Interestingly, Spira et al [37] observed that the pirin levels returned to normal for ex-smokers, supporting observation in the present study that pirin is influenced by acute exposure to cigarette smoke.

## Conclusion

Our current study suggests that pirin expression in the airway epithelium is in response to the acute oxidative stress imposed by cigarette smoke. Our study also suggests that pirin over-expression in response to cigarette smoke may play a role in epithelial cell apoptosis, and one possible mechanism is by interacting with NF-κB to induce the expression of pro-apoptotic genes, in cells injured by cigarette smoke [38]. Theoretically, apoptosis of the bronchial epithelium in response to smoking is a delicate balancing act. On the one hand, it is probably necessary to prevent DNA damage and subsequent mutagenesis that leads to lung cancer. On the contrary, it can also lead to a loss of epithelial integrity followed by chronic inflammation that results in chronic bronchitis.
